# *Kaempferia parviflora* extract increases energy consumption through activation of BAT in mice

**DOI:** 10.1002/fsn3.144

**Published:** 2014-07-15

**Authors:** Susumu Yoshino, Minji Kim, Riyo Awa, Hiroshige Kuwahara, Yuriko Kano, Teruo Kawada

**Affiliations:** 1 Research Center, Maruzen Pharmaceuticals Co., Ltd.Fukuyama, Hiroshima, 729-3102, Japan; 2 Laboratory of Molecular Function of Food, Division of Food Science and Biotechnology, Graduate School of Agriculture, Kyoto UniversityUji, Kyoto, 611-0011, Japan; 3 Laboratory of Nutrition Chemistry, Faculty of Home Economics, Kobe Women's UniversitySuma-ku, Kobe, Hyogo, 654-8585, Japan; 4 Research Unit for Physiological Chemistry, C-PIER, Kyoto UniversityKyoto, 606-8501, Japan

**Keywords:** cAMP, *Kaempferia parviflora*, oxygen consumption, UCP1

## Abstract

*Kaempferia parviflora* (KP) is a member of the ginger family and is known in Thailand as Thai ginseng, Krachai Dam or Black Ginger. The*K. parviflora* extract (KPE) was previously reported to have a number of physiological effects; however, the antiobesity effects of KPE and its mechanisms remain to be elucidated. In this study, we conducted KPE feeding experiments (low dose: 0.5% KPE, high dose: 1.0% KPE) in mice to examine the antiobesity effects. For both 0.5% KPE and 1.0% KPE, 7 weeks’ feeding of KPE contained in a high-fat diet (HFD) significantly decreased body weight gain, intraabdominal fat accumulation, and plasma triglyceride and leptin levels. Concurrently, KPE administration increased oxygen consumption in mice fed on a HFD. We also found that 1.0% KPE feeding significantly increased the uncoupling protein 1 (UCP1) expression in brown adipose tissue (BAT). Moreover, KPE administration increased urinary noradrenaline secretion levels. These results demonstrate that KPE promotes energy metabolism by activation of BAT, at both doses and up-regulation of UCP1 protein at a high dose. Although numerous challenges remain, the present study demonstrated that KPE suppresses HFD-induced obesity through increased energy metabolism.

## Introduction

Obesity is a serious worldwide epidemic and has necessitated various obesity-related studies; however, at present, most weight-loss methods involve exercise therapy and dieting. Thus, the search for food products that can reduce obesity holds great promise.

*Kaempferia parviflora* (KP) is a member of the ginger family and is known in Thailand as Thai ginseng, Krachai Dam or Black Ginger. The*K. parviflora* extract (KPE) was previously demonstrated to have a number of physiological effects, including antioxidant, antiinflammatory, antiallergic, antitumor, cardioprotective, and antibacterial activities (Rujjanawate et al. 2005; Tewtrakul and Subhadhirasakul 2008; Kusirisin et al. 2009; Tep-areenan et al. 2010). However, there are few reports documenting the antiobesity effects of KPE. Thus, we examined the effects of KPE on obesity in mice. The rhizomes of KP from Thailand were washed thoroughly in water, dried and powdered. The rhizome powder was extracted with 50% ethanol, evaporated in vacuo and freeze-dried to obtain a dry extract which from now on is referred to as KPE.

**Figure 1:**
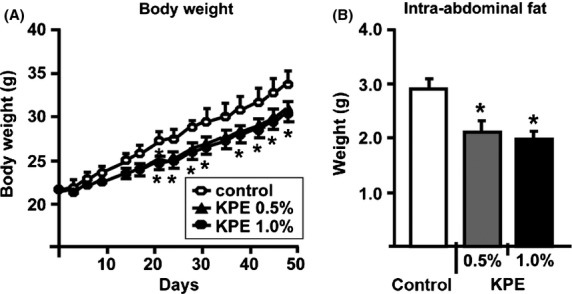
KPE suppresses body weight gain and intraabdominal fat in C57BL/6J mice. Temporal changes in body weight (A) and amount of intraabdominal fat (B) of C57BL/6J mice with and without KPE (0.5% and 1.0%) treatment for 7 weeks under HFD feeding. These procedures of animal experiments were approved by the Animal Care and Use Committee at Maruzen Pharmaceuticals Co., Ltd. (FD-A0012). Each bar represents the mean ± SE (*n* = 8). *P < 0.05 compared with the untreated control group. KPE,*K. parviflora* extract; HFD, high-fat diet.

**Figure 2:**
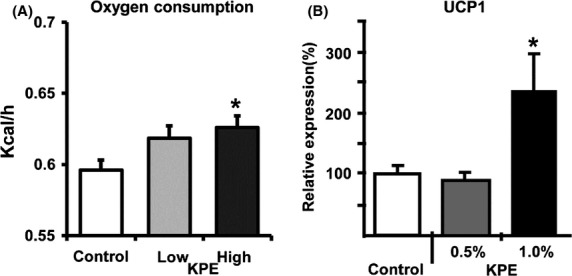
KPE increases oxygen consumption and UCP1 expression levels of BAT in C57BL/6J mice. (A) Oxygen consumption of C57BL/6J mice orally administered KPE (0, 0.035 or 0.105 mg/g body weight). Oxygen consumption was measured every 8 min for 24 h using an indirect calorimetric system (Oxymax; Columbus Instruments, Columbus, OH). Each bar represents the mean ± SE (*n* = 8). *P < 0.05 compared with the untreated control group. (B) UCP1 expression levels of BAT mitochondria in C57BL/6J mice with and without KPE (0.5% and 1.0%) treatment for 7 weeks under HFD feeding. BAT mitochondria were isolated and purified as reported previously, and the total protein content in BAT mitochondria was measured with DC Protein Assay Kit (Bio-Rad, CA). UCP1 expression levels in the mitochondrial fraction were measured by western blotting analysis. A mitochondrial extract from BAT was subjected to SDS-PAGE. UCP1 expression levels in the untreated control group were set at 100%, and relative UCP1 expression levels were presented as the fold induction relative to that of the untreated control group. Each bar represents means ± SE (*n* = 8). *P < 0.05 compared with untreated control group. UCP1, uncoupling protein 1; BAT, brown adipose tissue; KPE,*K. parviflora* extract.

C57BL6J male mice were fed a high-fat diet (HFD; 60 kcal % fat) containing KPE (0.5 or 1.0%) for 7 weeks. In this study, both 0.5% KPE and 1.0% KPE administration significantly suppressed body weight gain and intraabdominal fat (Fig.1A and B) and decreased serum triglyceride and leptin levels (Table S1). Leptin levels are known to correlate with weight and amount of visceral fat in mice and humans (Shimizu et al. 1997; Ahren 1999); the results from the present study are consistent with these previous reports (Fig.1 and Table S1). In these experiments, there were no differences in total calories consumed during the study period (Fig. S1), suggesting that the reason for the loss of weight and intraabdominal fat was an increase in energy consumption. To explore this possibility, we examined changes in oxygen consumption using the previous method (Goto et al. 2012) after oral administration of KPE (low dose; 0.035 mg/g, high dose; 0.105 mg/g) (Fig.2A). Oxygen consumption after administration was significantly higher in mice treated with KPE. This result indicates that KPE increases oxygen consumption, thus this is one mechanism contributing to its antiobesity effects.

In rodents, brown adipose tissue (BAT) is an important organ for energy expenditure through thermogenesis (Lowell et al. 1993). Uncoupling protein-1 (UCP1) is considered to play an important role in thermogenesis in BAT (Rothwell and Stock 1979; Feldmann et al. 2009; Lee et al. 2012). UCP1 generates heat by leaking proteins across the mitochondrial inner membrane, thus uncoupling oxidative phosphorylation without ATP production. Ablating UCP1 causes cold sensitivity and obesity in mice. Thus, UCP1 in BAT is essential for thermogenesis (Rothwell and Stock 1979; Lowell et al. 1993; Feldmann et al. 2009; Lee et al. 2012). Previous studies showed that BAT helps improve glucose metabolism and that it is critical for the normal cerebral functioning of leptin, an appetite-suppressing hormone secreted from white adipose tissue (WAT) (Okamatsu-Ogura et al. 2011). This suggests that BAT is an extremely important organ for antiobesity mechanisms. Recently, it was reported that in addition to human fetuses and newborns, BAT also exists in human adults (Cypess et al. 2013; Saito 2013), and that BAT-like cells (beige cells, also known as “BRIGHT” cells) that are formed in WAT also exist (Wu et al. 2012; Lidell et al. 2013). As BAT is present in humans as well as rodents, it shows promise as a target site for reducing obesity through thermogenesis. Therefore, we examined the effects of KPE administration on UCP1 expression in BAT using previously used methods (Cannon and Lindberg 1979; Kawada et al. 1991) to explore possible mechanisms of the antiobesity effects. In the present study, while we found that administration of 0.5% KPE for 7 weeks had no effect on UCP1 expression levels in BAT, 1.0% KPE significantly increased these levels (Fig.2B). These results demonstrate that KPE promotes energy metabolism by activation of BAT with a dose-dependent increase in UCP1 expression.

**Figure 3:**
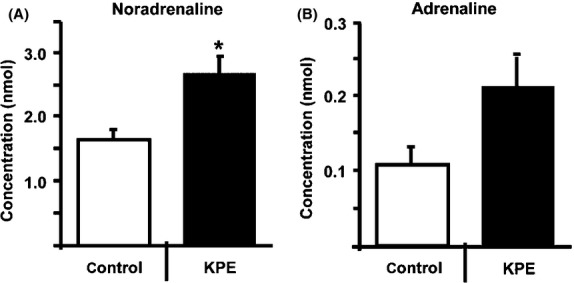
KPE increases urinary noradrenaline secretion in C57BL/6J mice. Urinary noradrenaline (A) and adrenaline (B) secretion in C57BL/6J mice with 0.5% KPE treatment (*n* = 8) and without KPE treatment (*n* = 6) for 2 weeks under HFD feeding. Urinary adrenaline and noradrenaline levels were measured by HPLC analysis. These procedures were aproved by the Institutional Animal Care and Use Committee of Kobe Women's University, Faculty of Home Economics (A316). Each bar represents means ± SE *P < 0.05 compared with untreated control group. KPE,*K. parviflora* extract.

UCP1 expression is regulated by sympathetic nerve activity (Mory et al. 1984; Feldmann et al. 2009). We hypothesized that KPE activates the sympathetic nerves. To test this hypothesis, we measured urinary concentrations of adrenaline and noradrenaline using the previously used method (Davidson and Fitzpartrick 1985), which are secreted upon sympathetic nerve activation. In this study, 2 weeks administration of 0.5% KPE significantly increased urinary noradrenaline secretion in C57BL/6J mice (Fig.3A). The results of the present experiment suggest that KPE feeding promoted noradrenaline secretion, thereby increasing triglyceride decomposition and leading to a decrease in serum triglyceride levels. It is known that adrenaline and noradrenaline cause an increase in cyclic adenosine monophosphate (cAMP) levels in adipocytes, and that cAMP regulates UCP1 expression via PKA (Cannon and Lindberg 1979). These results suggest that KPE consumption has antiobesity effects.

In the present study, both 0.5% KPE and 1.0% KPE feeding decreased body weight gain, intraabdominal fat accumulation, and plasma triglyceride and leptin levels. In addition, KPE promoted noradrenaline secretion. Moreover, 1.0% KPE feeding significantly increased UCP1 expression levels in BAT, whereas 0.5% KPE feeding had no effect. These results indicate that at a low dose (KPE 0.5%) KPE promotes energy consumption by activating BAT without increasing UCP1 expression, through increased cellular cAMP levels via enhanced noradrenaline secretion. At a high dose (KPE 1.0%), KPE promotes energy consumption by activation of BAT with an up-regulation of UCP1 protein. Although a number of aspects require clarification, such as identifying the active components in KPE and the mechanisms responsible for increasing sympathetic nerve activity, the present study demonstrated KPE to be a potentially useful food product for curbing and reducing obesity.

## Conflict of Interest

None declared.

## Supporting Information

**Table S1.** Plasma parameters assessed in C57BL/6J mice. Blood samples were collected from C57BL/6J mice with (0.5% or 1.0% KPE) and without (HFD) KPE treatment for 7 weeks. Glucose, triglycerides, free fatty acids, insulin and leptin levels in the blood were measured. Values are means ± SE (*n* = 8) **P* < 0.05 versus HFD control group. **Figure S1.** Total calory intake of C57BL/6J mice on HFD. Total calory intake of C57BL/6J mice with (0.5% or 1.0% KPE) and without (Control; HFD) during 7 weeks. Values are means ± SE (*n* = 8). **Figure S2.** Western blot analysis of UCP1 in BAT. Two micrograms of mitochondrial extract from BAT of control C57BL/6J mice or mice treated with KPE (0.5% and 1.0%) for 7 weeks under HFD feeding were subjected to SDS-PAGE. UCP1 was immunodetected using a UCP1-specific antibody. Relative control; same bach mitochondrial proteins in BAT of mice fed a standard diet.

## References

[b1] Ahren B (1999). Plasma leptin and insulin in C57BL/6J mice on a high-fat diet: relation to subsequent changes in body weight. Acta Physiol. Scand.

[b2] Cannon B, Lindberg O (1979). Mitochondria from brown adipose tissue: isolation and properties. Methods Enzymol.

[b3] Cypess AM, White AP, Vernochet C, Schulz TJ, Xue R, Sass CA (2013). Anatomical localization, gene expression profiling and functional characterization of adult human neck brown fat. Nat. Med.

[b4] Davidson DF, Fitzpatrick J (1985). A simple, optimized and rapid assay for urinary free catecholamines by HPLC with electrochemical detection. Ann. Clin. Biochem.

[b5] Feldmann HM, Golozoubova V, Cannon B, Nedergaard J (2009). UCP1 ablation induces obesity and abolishes diet-induced thermogenesis in mice exempt from thermal stress by living at thermoneutrality. Cell Metab.

[b6] Goto T, Teraminami A, Lee JY, Ohyama K, Funakoshi K, Kim YI (2012). Tiliroside, a glycosidic flavonoid, ameliorates obesity-induced metabolic disorders via activation of adiponectin signaling followed by enhancement of fatty acid oxidation in liver and skeletal muscle in obese-diabetic mice. J. Nutr. Biochem.

[b8] Kawada T, Sakabe S, Aoki N, Watanabe T, Higeta K, Iwai K (1991). Intake of sweeteners and pungent ingredients increases the thermogenin content in brown adipose tissue of rat. J. Agric. Food Chem.

[b9] Kusirisin W, Srichairatanakool S, Lerttrakarnnon P, Lailerd N, Suttajit M, Jaikang C (2009). Antioxidative activity, polyphenolic content and anti-glycation effect of some Thai medicinal plants traditionally used in diabetic patients. Med. Chem.

[b10] Lee JY, Takahashi N, Yasubuchi M, Kim YI, Hashizaki H, Kim MJ (2012). Triiodothyronine induces UCP-1 expression and mitochondrial biogenesis in human adipocytes. Am. J. Physiol. Cell Physiol.

[b11] Lidell ME, Betz MJ, Dahlgvist Leinhard O, Heglind M, Elander L, Slawik M (2013). Evidence for two types of brown adipose tissue in humans. Nat. Med.

[b12] Lowell BB, S-Susulic V, Hamann A, Lawitts JA, Himms-Hagen J, Boyer B (1993). Development of obesity in transgenic mice after genetic ablation of brown adipose tissue. Nature.

[b13] Mory G, Bouillaud F, Combes-George M, Ricguier D (1984). Noradrenaline controls the concentration of the uncoupling protein in brown adipose tissue. FEBS Lett.

[b14] Okamatsu-Ogura Y, Nio-Kobayashi J, Iwanaga T, Terao A, Kimura K, Saito M (2011). Possible involvement of uncoupling protein 1 in appetite control by leptin. Exp. Biol. Med.

[b15] Rothwell NJ, Stock MJ (1979). A role for brown adipose tissue in diet-induced thermogenesis. Nature.

[b16] Rujjanawate C, Kanjyanapothi D, Amornlerdpison D, Pojanagaroon S (2005). Anti-gastric ulcer effect of*Kaempferia parviflora*. J. Ethnopharmacol.

[b17] Saito M (2013). Brown adipose tissue as a regulator of energy expenditure and body fat in humans. Diabetes Metab. J.

[b18] Shimizu H, Shimomura Y, Hayashi R, Ohtani K, Sato N, Futawatari N (1997). Serum leptin concentration is associated with total body fat mass, but not abdominal fat distribution. Int. J. Obes. Relat. Metab. Disord.

[b20] Tep-areenan P, Sawasdee P, Randall M (2010). Possible mechanisms of vasorelaxation for 5,7-dimethoxyflavone from*Kaempferia parviflora* in the rat aorta. Phytother. Res.

[b19] Tewtrakul S, Subhadhirasakul S (2008). Effects of compounds from*Kaempferia parviflora* on nitric oxide, prostaglandin E2 and tumor necrosis factor-alpha productions in RAW264.7 macrophage cells. J. Ethnopharmacol.

[b7] Wu J, Boström P, Sparks LM, Ye L, Choi JH, Giang AH (2012). Beige adipocytes are a distinct type of thermogenic fat cell in mouse and human. Cell.

