# Supplementary material for: Kaempferia parviflora extract increases energy consumption through activation of BAT in mice
Source: Food Sci Nutr. 2014 Jul 15;2(6):634–7. doi: 10.1002/fsn3.144 (PMC4256566; doi:10.1002/fsn3.144)

**Supplemental data**

**Table 1. Plasma parameters assessed in C57BL/6J mice.**

Blood samples were collected from C57BL/6J mice with (0.5% or 1.0% KPE) and without (HFD) KPE treatment for 7 weeks. Glucose, triglycerides, free fatty acids, insulin and leptin levels in the blood were measured. Values are means ± S.E. (n=8). * p<0.05 vs. HFD control group.


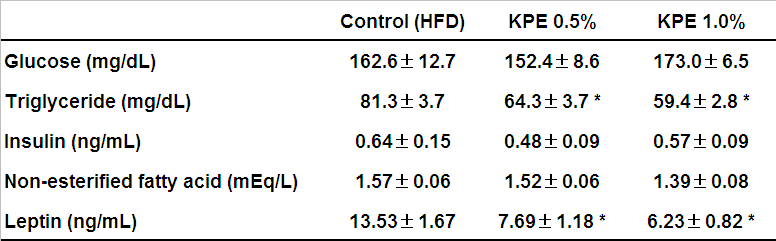


**Figure 1. Total calory intake of C57BL/6J mice on HFD**

Total calory intake of C57BL/6J mice with (0.5% or 1.0% KPE) and without (Control; HFD) during 7 weeks. Values are means ± S.E. (n=8).

**
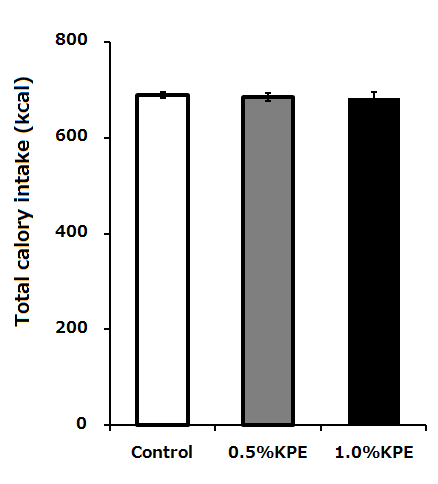
**

**Figure 2. Western blot analysis of UCP1 in BAT.**

Two micrograms of mitochondrial extract from BAT of control C57BL/6J mice or mice treated with KPE (0.5 and 1.0%) for 7 weeks under HFD feeding were subjected to SDS-PAGE. UCP1 was immunodetected using a UCP1-specific antibody. Relative control; same bach mitochondrial proteins in BAT of mice fed a standard diet.


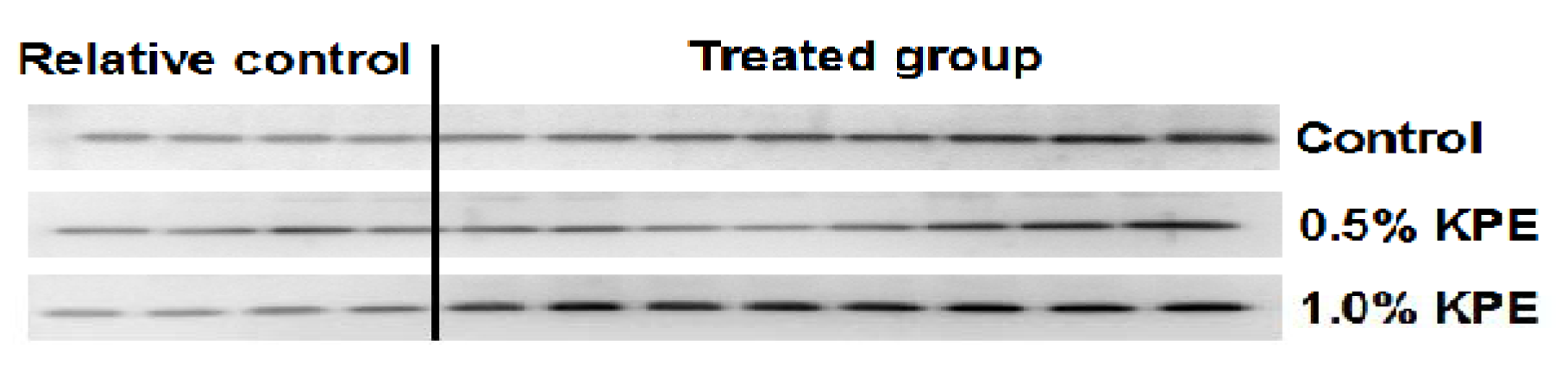

Supplement: Supplementary file 1 — Table S1. Plasma parameters assessed in C57BL/6J mice. Blood samples were collected from C57BL/6J mice with (0.5% or 1.0% KPE) and without (HFD) KPE treatment for 7 weeks. Glucose, triglycerides, free fatty acids, insulin and leptin levels in the blood were measured. Values are means ± SE (n = 8) *P < 0.05 versus HFD control group. Figure S1. Total calory intake of C57BL/6J mice on HFD. Total calory intake of C57BL/6J mice with (0.5% or 1.0% KPE) and without (Control; HFD) during 7 weeks. Values are means ± SE (n = 8). Figure S2. Western blot analysis of UCP1 in BAT. Two micrograms of mitochondrial extract from BAT of control C57BL/6J mice or mice treated with KPE (0.5% and 1.0%) for 7 weeks under HFD feeding were subjected to SDS-PAGE. UCP1 was immunodetected using a UCP1-specific antibody. Relative control; same bach mitochondrial proteins in BAT of mice fed a standard diet. [file fsn30002-0634-sd1.docx]
